# 941. Isavuconazole Prophylaxis Against Invasive Fungal Infection: A Pooled Analysis with a Comparison of Posaconazole Delayed-release Tablet Prophylaxis

**DOI:** 10.1093/ofid/ofab466.1136

**Published:** 2021-12-04

**Authors:** Chaeryoung Lee, Sung Kwan Hong, Jong Hun Kim

**Affiliations:** 1 CHA Bundang Medical Center, Seongnam, Kyonggi-do, Republic of Korea; 2 Korea University, Seoul, Seoul-t’ukpyolsi, Republic of Korea

## Abstract

**Background:**

There are limited data regarding the use of isavuconazole as primary antifungal prophylaxis against invasive fungal infection (IFI) among immunocompromised patients. Therefore, the purpose of this study was to assess efficacy and breakthrough IFIs of isavuconazole prophylaxis by a pooled analysis of the reported cases of isavuconazole prophylaxis with a comparison of cases of posaconazole delayed-release tablet prophylaxis.

**Methods:**

Pubmed was searched for English-written articles published up to April 2021. Studies that reported cases of primary antifungal prophylaxis with isavuconazole or posaconazole delayed-release tablet in adults ≥ 18 years were reviewed. Breakthrough IFI was defined as the occurrence of proven or probable IFI while on prophylaxis.

**Results:**

For overall isavuconazole prophylaxis, a total of 818 courses of prophylaxis was identified from 12 studies. Breakthrough IFIs were noted in 41 patients. The median duration of isavuconazole prophylaxis of these patients before the diagnosis of IFI was 17 days. The most common breakthrough IFI was candidiasis (34.1%), followed by aspergillosis (24.4%) and mucormycosis (12.2%). Sixteen patients died (39.0%). Among patients with hematologic malignancies or hematopoietic stem cell transplantation, isavuconazole prophylaxis (404 courses) was compared with posaconazole delayed-release tablet prophylaxis (1952 courses). The incidence rate of breakthrough IFIs was higher in the cohort of isavuconazole prophylaxis (24 patients of 404 courses) than in the cohort of posaconazole delayed-release tablet prophylaxis (44 patients of 1952 courses). Aspergillosis (40.9%) was the most common breakthrough IFI in the cohort of isavuconazole prophylaxis among patients with hematologic malignancies or hematopoietic stem cell transplantation, followed by candidiasis (27.3%) and mucormycosis (18.2%).

**Conclusion:**

Although isavuconazole is licensed to treat aspergillosis and mucormycosis, breakthrough IFIs including aspergillosis, mucormycosis, and candidiasis may occur while on isavuconazole prophylaxis. Therefore, further studies are needed to define the benefits and risks of isavuconazole prophylaxis.

**Disclosures:**

**All Authors**: No reported disclosures

